# Rapid, low cost and sensitive detection of Calreticulin mutations by a PCR based amplicon length differentiation assay for diagnosis of myeloproliferative neoplasms

**DOI:** 10.1186/s12881-019-0819-6

**Published:** 2019-06-27

**Authors:** Ngo Tat Trung, Dao Thanh Quyen, Nghiem Xuan Hoan, Dao Phuong Giang, Tran Thi Huyen Trang, Thirumalaisamy P. Velavan, Mai Hong Bang, Le Huu Song

**Affiliations:** 1 Centre for Genetic Consultation and Cancer Screening, 108 Institute of Clinical Medical and Pharmaceutical Sciences, Hanoi, Vietnam; 2 Vietnamese - German Center for Medical Research, 108 Institute of Clinical Medical and Pharmaceutical Sciences, No 1, Tran Hung Dao Street, Hai Ba Trung District, Hanoi, Vietnam; 3 Department of Molecular Biology, 108 Institute of Clinical Medical and Pharmaceutical Sciences, Hanoi, Vietnam; 4 Faculty of Gastroenterology, 108 Institute of Clinical Medical and Pharmaceutical Sciences, Hanoi, Vietnam; 5 Institute of Tropical Medicine, Universitätsklinikum Tübingen, Tübingen, Germany; 6 Faculty of Tropical and Infectious Diseases, 108 Institute of Clinical Medical and Pharmaceutical Sciences, Hanoi, Vietnam

**Keywords:** Myeloproliferative neoplasms, *JAK2* V617F, *CALR* mutations

## Abstract

**Background:**

Calreticulin (*CALR*) gene mutations are currently recommended as biomarkers in diagnosis of patients with myeloproliferative neoplasms (MPN) with *Jak2* V617F negative phenotype. Our aim was to establish a rapid, low cost and sensitive assay for identification of *CALR* gene mutations and to validate the diagnostic performance of the established assay in a patient cohort with different clinical MPN phenotypes.

**Methods:**

One hundred five Philadelphia-negative MPN patients, including polycythemia vera (PV), essential thrombocythaemia (ET), and primary myelofibrosis (PMF) were initially screened for *JAK2* mutations by amplification-refractory mutation system (ARMS-PCR) methodology and were further subjected to detection of *CALR* gene mutations by our in-house assay, a PCR based amplicon length differentiation assay (PCR-ALDA). The PCR-ALDA methodology was compared with real time PCR and Sanger sequencing methods. Furthermore, the analytical sensitivity of the assay was established.

**Results:**

PCR - ALDA approach was able to detect and discriminate the pseudo-positive samples containing more than 1% *CALR* mutant alleles. *CALR* mutations were not detected in 63 *Jak2* V617F positive cases in all three methods. In contrast, amongst 42 *Jak2* V617F negative cases, both PCR-ALDA and Sanger sequencing coherently identified 12 *CALR* mutants compared to 10 *CALR* mutants detected by real-time PCR method.

**Conclusion:**

PCR-ALDA can be utilized as an easy-to-use, rapid, low cost and sensitive tool in the detection of *CALR* mutations in Philadelphia-negative MPN patients.

**Electronic supplementary material:**

The online version of this article (10.1186/s12881-019-0819-6) contains supplementary material, which is available to authorized users.

## Background

The myeloproliferative neoplasms (MPN) are clonal disorders of hematopoietic progenitors that includes the classical chronic myeloid leukemia (CML), polycythemia vera (PV), essential thrombocythemia (ET), primary myelofibrosis (PMF), chronic eosinophilic leukemia (CEL), chronic myelomonocytic leukemia (CMML), and systemic mastocytosis [1]. Three distinct phenotypes of PV, ET and PMF mainly constitute to Philadelphia-negative myeloproliferative syndromes [2].

Genetic alterations in both Janus Kinase 2 (*Jak2*) and thrombopoietin receptor myeloproliferative leukemic (*MPL)* virus oncogenes serve as molecular targets in the diagnosis of MPN [3]. Approximately, 60% of patients with MPN carry a non-synonymous substitution (V617F) in exon14 of the *Jak2* [4]. Among patients with distinct clinical phenotypes, 90% with PV and 60% with ET and PMF also carry these discrete gene mutations. Likewise, other *Jak2* variants in exon 10 and 12 [4, 5] and non-synonymous MPL variant in exon 10 (W515 L and W515K) [5, 6] were also reported to occur among 5% of patients with MPN.

Albeit, *Jak2/MPL* genetic variants essentially contribute in the diagnoses of MPN, a significant number of MPN patients can be missed in genetic screening due to the absence of these mutations. Nevertheless, 80–85% of *Jak2* V617F negative MPN patients can be diagnosed with a recently discovered distinct frame-shift mutation in exon 9 of the calreticulin (*CALR*) gene [7, 8] which weakens Ca2 + binding affinity of CALR [9, 10]. Since its discovery, *CALR* domain serve as valuable molecular target for the diagnosis of clonal MPNs and since then World Health Organization (WHO) has revised the diagnoses algorithm for patients with Philadelphia-negative MPNs [1, 11].

Until date, several *CALR* mutations had been described. However, two of those namely, type − 1 (52 bp deletion) and a type-2 (5 bp insertion) mutations account for 85% of *Jak2* V617F Philadelphia-double negative MPNs [7, 8]. Until date, consensus has yet not been established for use of a single given methodology for *CALR* genotyping [12–14]. Current *CALR* genetic mutation screening methods include high resolution melting curve analysis (HRMA), direct Sanger sequencing and real-time PCR based techniques that utilize allele specific probes. All current available assays in the detection of *CALR* mutations are arguable, either on cost, sensitivity and/or specificity.

This study aimed to establish simultaneous detection of *CALR* type-1 and *CALR* type-2 mutations following a PCR based method to differentiate the alleles based on amplicon size on a normal agarose gel. This established PCR based amplicon length differentiation assay (PCR-ALDA) was further validated and evaluated with other available assays for their diagnostic performance in a cohort of Vietnamese patients with MPN devoid of JAK2 V617F mutation.

## Methods

### Patient population

**Fig. 1:**
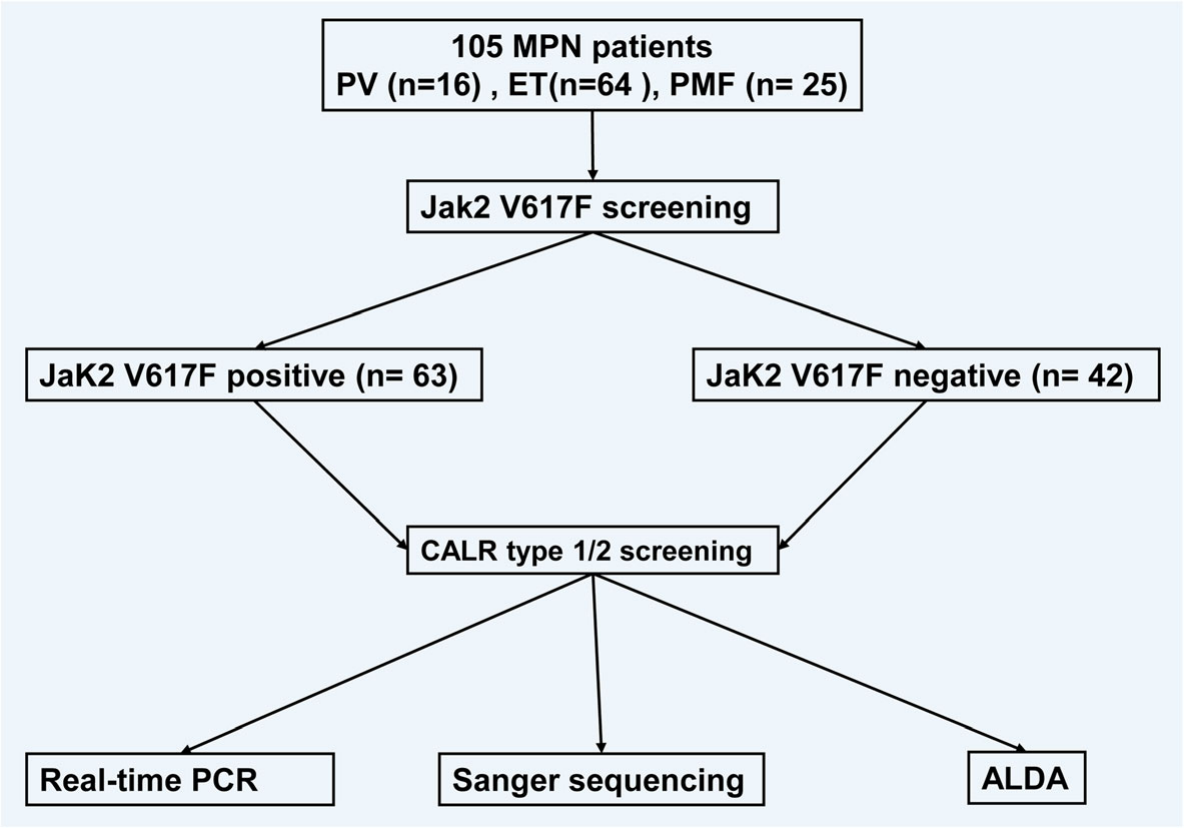
Study design. All recruited MPN patients were initially screened for BCR-ABL transcripts to differentiate BCR-ABL positive or negative MPN patients using real time PCR. All 105 MPN patients BCR-ABL transcripts negative were subjected to an ARMS-PCR method to screen for the presence or absence of the Jak2 V617F mutation. Irrespective of the presence or absence of wildtype or mutant, all 105 MPN patients were subjected to an in-house developed PCR based protocol (ALDA -amplicon length differentiation assay) for simultaneous detection of CALR type-1 and CALR type-2 mutations. Furthermore the ALDA assay was comparatively evaluated with Sanger sequencing and other available real-time PCR methodologies as described by Zinke et. al [13]

A total 105 Philadelphia-negative MPN patients were recruited at 108 Military Central Hospital from January 2017 until November 2018 and classified based on clinical phenotype as PV (*n* = 16), ET (*n* = 64), and PMF (*n* = 25). The recently revised WHO diagnoses criterion for MPNs was used to define PV, ET and PMF cases [11]. The entire study flow is illustrated in Fig. 1. In brief: All recruited MPN patients were initially screened for BCR-ABL transcripts to differentiate BCR-ABL positive or negative MPN patients using real time PCR. All 105 MPN patients BCR-ABL transcripts negative were subjected to an ARMS-PCR method to screen for the presence or absence of the Jak2 V617F mutation. Irrespective of the presence or absence of wildtype or mutant, all 105 MPN patients were subjected to an in-house developed PCR based protocol (ALDA -amplicon length differentiation assay) for simultaneous detection of CALR type-1 and CALR type-2 mutations. Furthermore the ALDA assay was comparatively evaluated with Sanger sequencing and other available real-time PCR methodologies as described by Zinke et.al [13].

Upon hospital admission, 1 ml of venous blood was collected and stored as 0.5 ml aliquot each until further use. The haematological profile of recruited patients is summarized in Table 1. One aliquot of 0.5 ml was utilized for RNA extraction, cDNA conversion followed by a real time PCR assay, whereas the other 0.5 ml aliquot of blood for DNA extraction and subsequent genotyping of *Jak2* V617F and *CALR* mutations. The utilized primer pairs in this study are provided in Additional file 1: Table S1.

**Table 1:** Baseline characteristics of all PMN patients and subgroups classified into ET, PMF and PV syndrome

Clinical characteristics	PMN (*n* = 105)	ET (*n* = 67)	PV (n = 16)	PMF (*n* = 22)	*P* ^a^
Gender - male (%)	77 (73.3)	50 (74)	10 (62.5)	17 (77.3)	NS
Age (years)	64 (16–96)	64 (16–96)	69 (29–86)	57 (29–85)	NS
WBC (× 10^3^/mL)	14.3 (1.9–87.2)	14.8 (6.8–66.5)	8.2 (5.2–31.9)	15.5 (1.9–87.2)	0.039
RBC (× 10^6^/mL)	4.8 (2.1–9.7)	4.5 (2.5–8.1)	5.7 (5.4–9.7)	4.5 (2.14–9.7)	0.0002
PLT(×10^3^/mL)	710 (45–2301)	820 (376–2301)	233 (150–803)	246 (45–1776)	6.15e-11
Hb (g/L)	138 (10.5–211)	132 (10.5–188)	185 (167–211)	125 (66–178)	2.17e-08

Abbreviation: Hb, hemoglobin; PLT, platelets; WBC, white blood cell; RBC, red blood cell; NS, not significant. Values given are medians and range. (^a^) in comparison between subgroups (ET, PV and PMF). *P* values were calculated by Chi-squared and Kruskal-Wallis test where appropriate

### Screening for *BCR-ABL* transcripts

The *BCR-ABL* transcripts was screened in patients peripheral blood using a standard assay [15]. In brief: 0.5 ml of total blood was mixed with 4.5 ml erythrocyte lysis buffer (20 mM Tris HCl pH 6.8; 5 mM MgCl2; 10 mM NaCl) and was centrifuged at 1500 rpm for 15 min at room temperature. The supernatant was discarded and 0.5 ml of TRIzol reagent (ThermoFisher Scientific Inc. Singapore) was suspended to the white blood cell (WBC) pellets. Following the manufacturers protocol, the total RNA was isolated and reconstituted in 50 μl diethylpyrocarbonate (DEPC) treated water. Approximately 500 ng of total RNA was reverse transcribed to cDNA using RevertAid First Strand cDNA Synthesis Kit (ThermoFisher Scientific Inc., Singapore), following the manufacturers instruction. Subsequently, the European Leukemia Net (ELN) standard real-time PCR protocol was followed for detecting *BCR-ABL* transcripts [15].

### Screening of *Jak2* V617F variant by ARMS-PCR

**Fig. 2:**
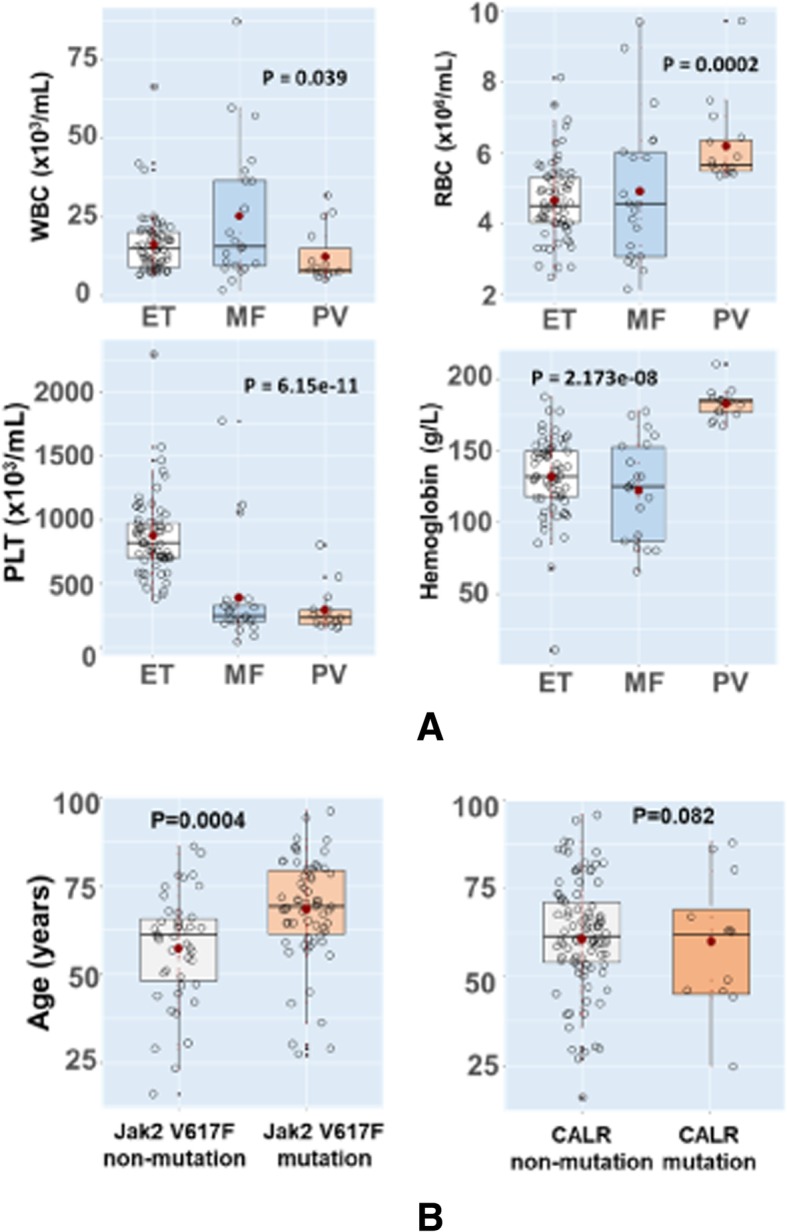
Distribution of haematological values and *Jak2* V617F, CALR mutant alleles in different study population. Panel **a**: Haematological parameters in: ET, PMF and PV subgroups. Panel **b**: Age distribution and *Jak2* V617F and *CALR* mutations; PLT: platelets; WBC: white blood cell; RBC: red blood cell; PLT: platelet; Hb: haemoglobin

The *Jak2* V617F mutation was screened following the amplification-refractory mutation system (ARMS-PCR) methodology [16]. In brief: four primer pairs were designed, two were outer primer pairs and two remained as targeted primer pairs that were allele specific. The outer primer pairs are Tr-Jak2-F: 5′- TCCTCAGAACGTTGATGGCAGTTG- 3′ and the TR-Jak2-R: 5′ -TCAGTTTCAAAAATACTTAACTCCTGT − 3′ and the amplification yield a product size of 405 bp. Two allele specific primer pairs targeting the mutant and major wild-type allele were also designed namely Tr-V617F-WT-F: 5′-GCATTTGGTTTTAAATTATGGAGTATTTG and Tr-V617F-MT-R: 5′-GTTTTACTTACTCTCGTCTCCACATAA-3′. The outer Tr-Jak2-F and Tr-V617F-MT-R would generate mutant amplicon of 279 bp, whereas the primer pairs Tr-Jak2-R and Tr- V617F-WT-F generate wild type amplicon of 181 bp. The locus, primer position and orientation of allele specific primers used in ARMS-PCR is illustrated in Fig. 2. The thermal cycling was with 32 cycles of 15 s at 95 °C denaturation; 30 s at 60 °C annealing and 30 s at 72 °C extension. The presence of mutation V167F yields an amplicon size of 279 bp, whereas wild type alleles indicated by an amplicon of 181 bp. The utilized ARMS-PCR methodology was further evaluated for their performance using standard/blinded samples provided by UK-NEQAS agency (http://www.ukneqasli.co.uk/eqa-pt-programmes/). Additionally, ARMS-PCR amplicons were subsequently subjected to Sanger sequencing to confirm the presence of the mutant or the major wildtype allele [17].

### *CALR* type-1 and -2 genotyping by amplicon length differentiation assay

**Fig. 3:**
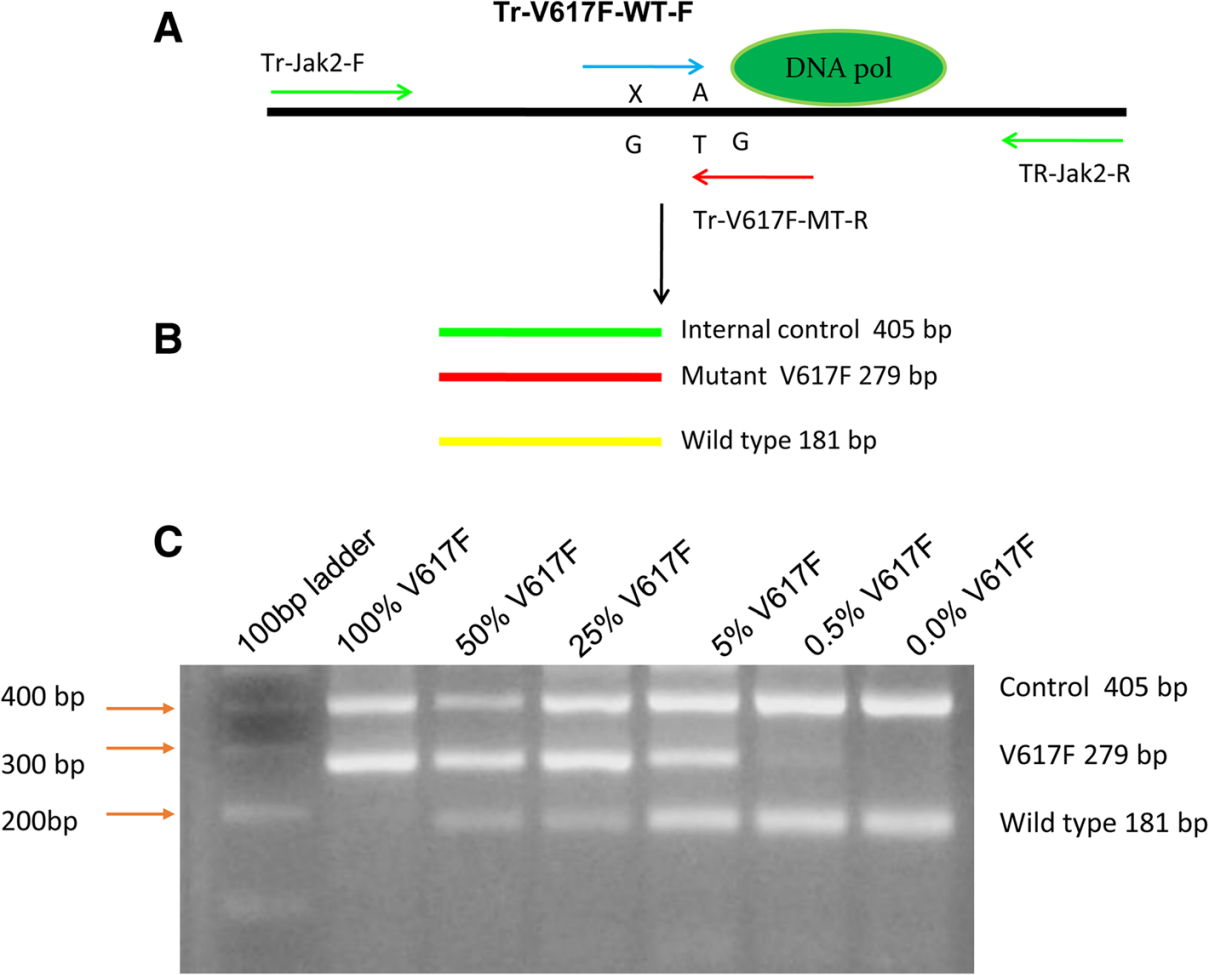
*Jak2* V617F screening by ARMS-PCR. Panel **a** with tetra primers: Jak2 V617F mutant allele amplified by primers (Tr-V617F-MT-R/Tr-Jak2-F) resulting in 279 bp product, whereas wild type allele amplified by primer pair (Tr-V617F-WT-F/TR-Jak2-R) resulting in 181 bp product. The amplicon produced by two outer primer pairs (Tr-Jak2-F/TR-Jak2-R) result in 405 bp product (internal control). Panel **b** Visualization on an agarose gel with dilution series to show resolution of tetra primer PCR product: Upper internal control (405 bp), V617F mutant (279 bp) and the *Jak2* wild type (181 bp). Detection is achieved in samples containing up to 0.5% V617F mutant allele

A PCR based method was developed for simultaneous detection of *CALR* type-1 (52 bp deletion) and a type-2 (5 bp-insertion). This established methodology was a PCR based protocol termed as amplicon length differentiation assay (PCR-ALDA). In brief, two primer pairs were used for this assay. The forward primer was chosen as previously described before in [7, 8] as TR-CALR-F: 5′-ACAAATGAAGGACAAACAGGACGA-3′, whereas the reverse primer was tailored [18] and was designed by Vector NTI.11.3 (ThermoFisher Scientific Inc. Singapore) using *CALR* gene sequences as obtained from NCBI database (GenBank Accession Nr. NG_029662) Q-Re-Del-CALR: 5′- CGGGGACATCTTCCTCCTCAT-3′. The primers utilized were able to efficiently and unbiasedly amplify both wild-type and known type-1 and -2 *CALR* mutant alleles. The concentration of individual primers is 0.25 pmol/μl. The reaction was run in an Eppendorf Master cycler (Eppendorf, Hamburg, Germany) with 32 cycles of 95 °C for 15 s; 60 °C for 30 s; 72 °C for 30 s. The amplicons were electrophoresed on a 2.5% agarose gel. A 171 base pair *CALR* wild-type allele against the deleted or inserted *CALR* alleles could be visualized on the agarose gel (Fig. 3).

### *CALR* type-1 and -2 genotyping by real time PCR

Two set of HPLC purified primer pairs as described by Zinke et al. [13] were synthesized (Integrated DNA Technology, IDT Singapore). The primer pairs for type − 1 mutation detection were CAL1-F: 5′-ACAGGACGAGGAGCAGATGA-3′ and CAL1-R: 5′-GGACATCTTCCTCCTCATCTTCCT-3′ that yield a 97 bp product whereas for type-2 mutation, the primer pairs were CAL2-F: 5′- GCTTAAGGAGGAGGAAGAAGACAA-3′; and CAL2-R: 5′-TGTCCTCATCATCCTCCGACAATT-3′, a 79 bp product for the type-2 mutation. The primer CAL1-F contains a G/T mismatch to further increase specificity, whereas the two fluorescent probe namely CAL1: FAM-TGTCCTCCTCATCCTCCTCATCCTCATCT-BHQ1 and CAL2-: FAM- CTGCCTCCTCCTCCTCTTTGCGT-BHQ1 were used to hybridize to the amplified products of the type − 1 deleted *CALR* or type − 2 inserted *CALR* respectively [13] The real-time PCR assay mixtures consisted of 7.5 μl Taqman real-time PCR master Mix (Qiagen, Hilden, Germany), 5 μl of genomic DNA or cDNA template, 1 ul of 5 pmol/ul of primers and 0.5 ul of 5 pmol/ul probe. The reactions were run in the Stratagene M3000P device (San Diego, CA, USA) with a pre-incubation step at 50 °C for 15 min, initial denaturation at 95^°^C for 5 min, followed by 45 cycles of 95^°^C for 15 s and 60^°^C for 60 s.

### Statistical analysis

All statistical analyses were performed using the R version 3.1.2 (http://www.r-project.org). Chi-square test was performed to compare categorical variables. Mann-Whitney U and Kruskal–Wallis tests were used to compare quantitative variables between two groups where appropriate. Revised WHO diagnostic criteria for MPN were used as reference to determine the diagnostic performance of the in-house procedure [11]. The statistical significance was set to a two-sided *P* value < 0.05.

## Results

### Baseline characteristics of MPN patients

Recruited patients were diagnosed based on clinical manifestations and haematological tests and subsequently classified into three groups: PV, ET and PM based on the revised WHO diagnostic criteria [11]. The screening of BCR-ABL transcripts performed to differentiate BCR-ABL positive or negative MPN patients confirmed that all investigated 105 MPN patients were BCR-ABL transcripts negative (Philadelphia chromosome negative). The baseline characteristics and the clinical parameters of the investigated study groups are presented in Table 1 and Fig. 2, respectively. A majority of patients were male (73%) with a median age of 64 years (range: 16–96). No difference in median age between three subgroups of MPNs (PV, ET and PM) was observed. Around 65 and 76% of all patients had WBC and Platelet (PLT) level above the upper normal limit (UNL), respectively. Whereas, 17% of those had increased levels of red blood cell (RBC) above UNL. PLT levels were significantly higher in ET patients than PV and PMF groups (*P* < 0.0001). Compared to ET and PM patients, the levels of Haemoglobin (Hb) and RBC in PV patients were significantly higher (P < 0.0001 and 0.0002, respectively); In contrast, WBC levels were lower (*P* = 0.039). The clinical features of study subjects were further characterized based on the presence of *Jak2* V617F and *CALR* mutations. *Jak2* V617F mutations were observed more frequently in older than younger patients (*P* = 0.0004). This finding indicates a possible senescence as a risk factor among these patients. In contrast, no significant contribution of age observed in the distribution of *CALR* mutations among the study groups. To understand effect of these two mutations on contributing to hematological characterizations, we analysed the association of *Jak2* V617F and *CALR* mutations with hematological measurements including WBC, RBC, PLT and Hb among all 105 patients. No significant association of hematological parameters either with *Jak2* V617F or *CALR* mutation was observed (*P* > 0.05).

### Analytical sensitivity of ARMS-PCR and the distribution of *Jak2* V617F

In order to determine the relative concentration at which ARMS-PCR can distinguish mutant allele fragments from wild-type, a stock DNA containing 100% V617F mutant fragment was diluted against wild type DNA extracted from healthy donor and a dilution series was made from 100, 50, 25, 5, 0.5, 0% until eventual negativity. All the dilutions were to 100 ng/ul and 5ul (equal to 500 ng) was used as template for each ARMS- PCR. Product mixtures were run through 1.2% agarose gel to resolve the mutant (297 bp) against wild type (181 bp) or internal control (405 bp). As observed in Fig. 3, ARMS-PCR could detect the presence of V617F mutant allele at a concentration of 0.5% and the threshold for such a limit of detection (LOD) was repeated several experiments by three independent technicians. Additionally, the external quality control for *Jak2* V617F identification was performed on blinded samples provided by the UK-NEQNAS that additionally acquired 100% accuracy (data not shown).

Subsequently, ARMS-PCR assay was used to screen for V617F, whereas Sanger sequencing was used for the screening of mutations within exon 12 of *Jak2*. We observed that 42 (40%) were negative for V617F mutant and 63 out of 105 (60%) MPN cases harboured *Jak2* V617F mutation. Among those, 60% (38/64) of ET patients, 44% (11/25) of PMF patients, and 87.5% (14/16) of PV patients had this mutation. Nevertheless, we did not detect Jak2 exon 12 mutation in any recruited subjects. Patients with and without Jak2 V617F mutation were then subjected to *CALR* mutations identification by Zinke’s method [13], Sanger sequencing and by established PCR based ALDA assay.

### PCR- ALDA and *CALR* genotyping

**Fig. 4:**
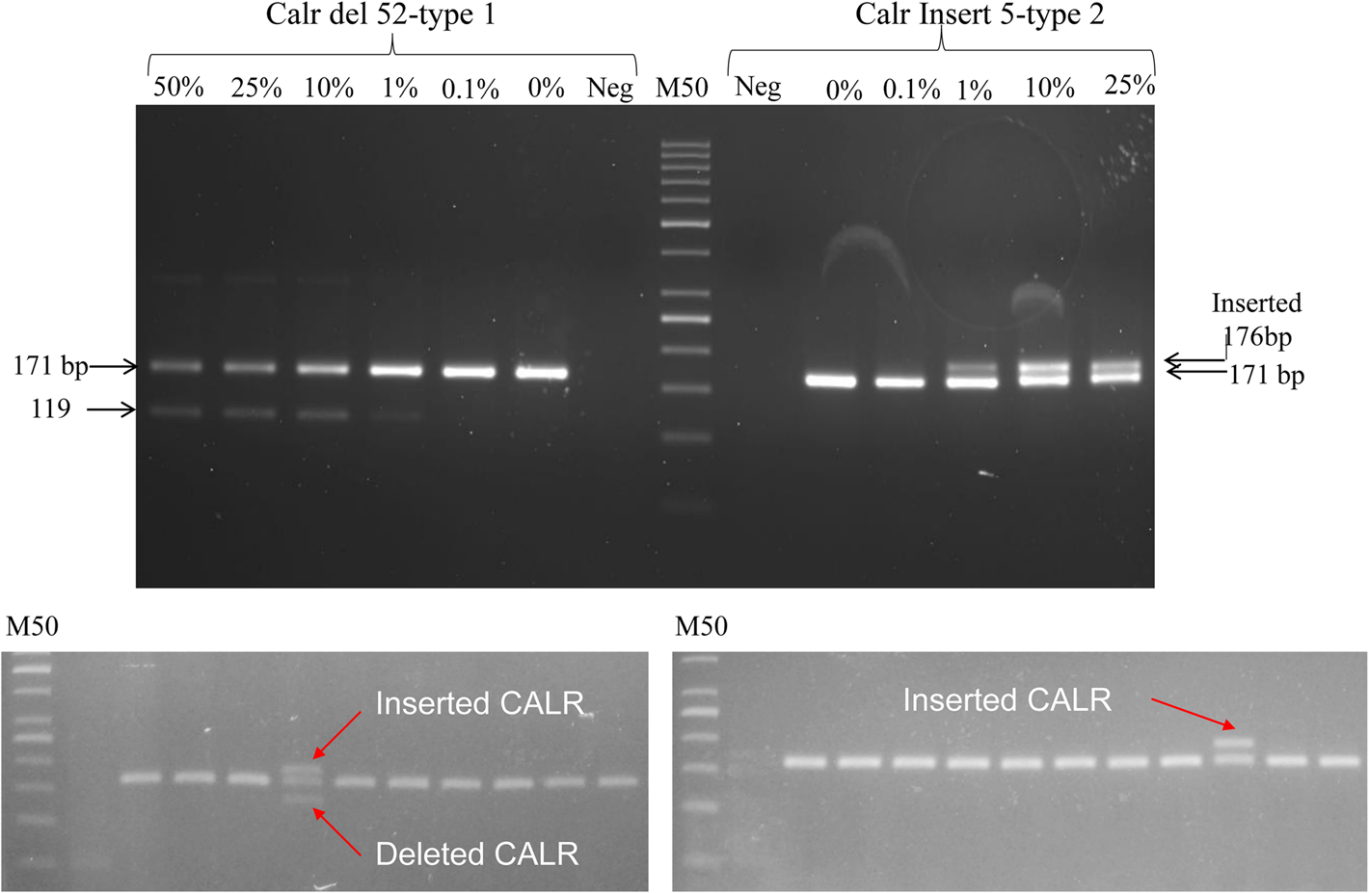
*CALR* genotyping and Limit of detection by PCR- ALDA. Dilution series were made by mixing 50% deleted *CALR* (upper left panel) or inserted *CALR* (upper right panel) containing DNA samples against wild type DNA. The dilution series were subjected to PCR-ALDA in which only one primer pair is used to unbiasedly amplify wild-type *CALR* allele and *CALR* mutant alleles. Subsequently, the reaction mixture was electrophoresed on a 2.5% agarose gel. The 171 bp-CALR-wild-type-allele against the 119 bp (type 1) 52 bp deleted *CALR* allele or 5 bp TTGTC inserted type − 2 mutation allele or other deleted and inserted alleles. Lower panel- *CALR* mutations in selected clinical samples: arrows indicate deletions or insertions

Both homo and heterozygous deletions can well be detected based on amplicon size differentiation. CALR del type-1 can well be distinguished by a 52 bp deletion with a product size of 119 bp (CALR del type 1 - homozygous mutant), a product size of 171 and 119 bp (CALR del type 1 - heterozygous mutant), whereas CALR type - 2 polymorphism well characterized by an insertion of 5 bp with a product size of 171 bp (CALR type 2 homozygous), a product size of 171 and 176 bp (CALR type 2 heterozygous mutant). In order to evaluate the detection limit of PCR-ALDA assay, we mixed DNA sample containing type − 1 or type − 2 *CALR* mutant DNA fragments against wild type DNA samples extracted from healthy donor” and a dilution series of 100 ng/μl containing 50, 25, 5, 0.5 and 0% *CALR* mutant alleles was utilized. These serial dilutions were treated as template for gel-based PCRs using one primer pairs that unbiasedly amplified both wild-type and mutant *CALR* alleles. Subsequently, the reaction mixture was electrophoresed on a 2.5% agarose gel to resolve the 171 base-pair-*CALR*-wild-type-allele against the deleted *CALR* type-1 or type − 2 insertion allele*.* As illustrated in the Fig. 3, PCR-ALDA approach could discriminate the pseudo-positive samples containing more than 1% *CALR* mutant alleles from the wild-type background. The results imply that the PCR-ALDA approach could detect more case carrying *CALR* mutations than Zinke’s Real-time PCR approach which was described as a deeper technical sensitivity at levels of 0.1% (Fig. 4).

### Evaluation of PCR-ALDA compared to sanger sequencing and real-time PCR

**Fig. 5:**
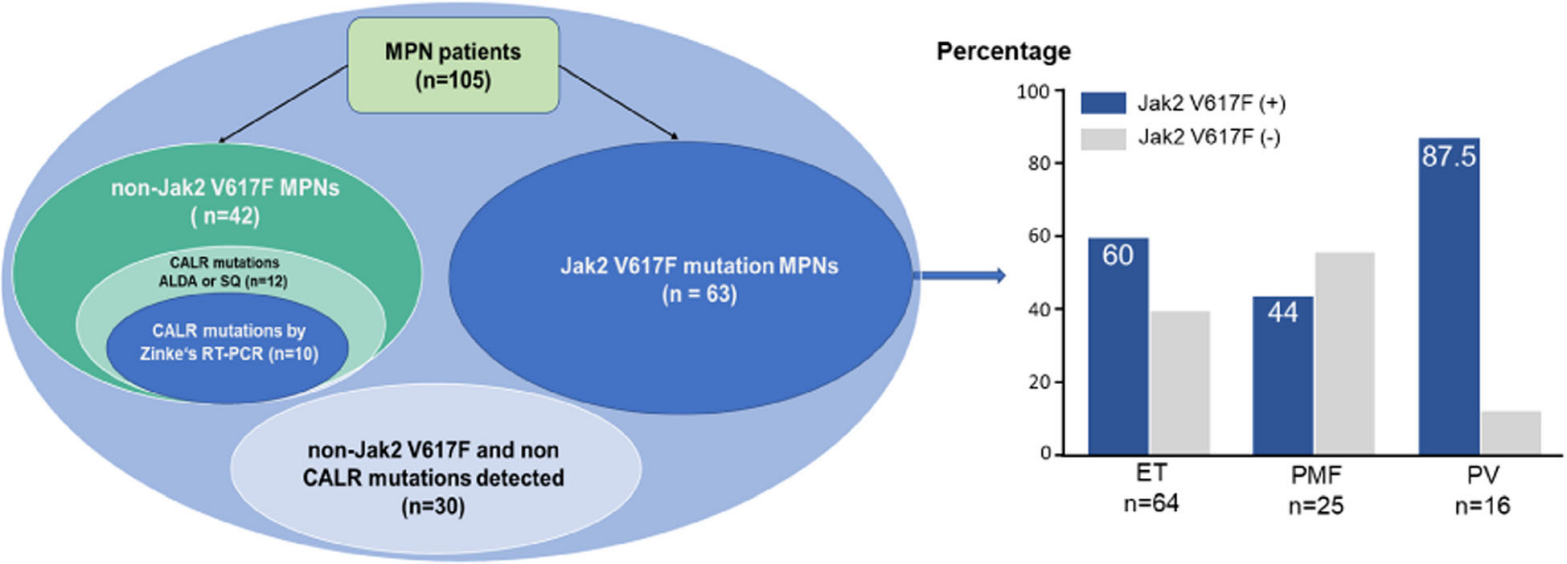
Evaluation of PCR-ALDA compared to Sanger sequencing and real-time PCR. Venn diagram and bar graph illustrating the distribution of Jak2 V617F and CALR mutations among 105 MPN patients. Screening for *Jak2* V617F was performed by ARMS-PCR and detection of *CALR* mutants by PCR-ALDA, real-time PCR (rt-PCR) and by direct Sanger sequencing (SQ)

To further evaluate the diagnostic performance of our PCR-ALDA approach, we subjected all 63 Jak2 V617F positive and 42 Jak2 negative DNA samples to PCR-ALDA, Zinke’s Real-time PCR and Sanger sequencing (Fig. 5). Our data revealed that no *CALR* mutant allele was detected in all 63 Jak2 V617F positive DNA samples by these methods. In contrast, amongst 42 Jak2 V617F negative cases, PCR-ALDA and Sanger sequencing equally identified 12 *CALR* mutant samples (Additional file 2: Figure S1), whereas only 10 mutant samples were detected by Zinke’s Real-time PCR (Additional file 3: Figure S2).

## Discussion

Accurate detection of disease-specific mutations facilitates precise diagnostics and better treatment regimens. In the scope of MPN, the best example is the *BCR-ABL* associated chronic myeloid leukemia (*BCR-ABL* positive CML) [11]. No other genetic lesions in MPN acquire a similar degree of diagnostic accuracy or therapeutic relevance like that of *BCR-ABL* positive CML. However, *Jak2* mutations are detected in significant portions of Philadelphia-negative MPN patients including PV, ET, PMF and in a minor part of the patients carrying *MPL* mutations. Therefore, both *Jak2* or *MPL* mutations are used as clonal markers in establishing the diagnosis of MPN [1, 11]. The fact is that not all MPN patients possess *Jak2* mutations and only minor proportion of MPN patients carries *MPL* mutations, indicating that the diagnostic value of *Jak2* or *MPL* mutations is limited by suboptimal sensitivity and specificity. The molecular diagnostic gap in *Jak2/MPL*-unmutated MPN patients is complimented by the recent discovery of *CALR* mutations in the majority of such cases [7, 8]. However, the described genetic lesions of *CALR* gene are very heterogenous that make a technical difficulty to gain a consensus approach for routine diagnosis of *CALR* mutations in clinical settings.

In this study, we used an ARMS-PCR based Jak2 V617F method to screen for the presence or absence of the *Jak2* V617F mutation in BCR-ABL transcript negative MPN patients. This assay is very clinically relevant in the context of acquiring an acceptable limit of detection (LOD) at 0.5%, which is comparable to the levels developed by other groups [19, 20]. At this level of LOD, we are able to identify 63 Jak2 V617F positive cases (60%) from 105 recruited MPN samples. The distribution of Jak2 mutation reported in this study was expectable as it falls in range similar to that of previously published data [3, 21]. The identification of *Jak2* V617F split our study cohort into two different subgroups: *Jak2* V617F positive group (Jak-pos) which is found in elder patients and characterized by higher levels of WBC, RBC and PLT but lower level of Hb compared to that of *Jak2* V617F negative (Jak-neg) group. This observation is similar to previous studies [22, 23]. Additionally, our *Jak2* V617F screening assay works very simple, without special requirement of equipment or personnel. However, we have not yet validated this assay for the purposes of quantitative monitoring like previous studies [19, 20], therefore, we only recommend to use our in-house assay for identification of *Jak2* V617F but not for quantification of *Jak2* mutant alleles in clinical practice.

As mentioned, the molecular diagnostic gap in *JAK2/MPL*-unmutated ET/PMF patients can be complemented by the identification of *CALR* gene mutations [7, 8]. Therefore, we proposed the PCR-ALDA approach for surveillance of these mutations. Our data revealed that PCR-ALDA approach is obviously acquired a limit of detection (LOD) at 1%. This quantitative level is not stronger than that of Zinke‘s [13] or the other approach [14]. Nevertheless, as the PCR-ALDA, Zinke’s approach and Sanger sequencing were comparatively co-applied to the same studied cohort, the PCR-ALDA assay has demonstrated a better diagnostic performance over that gained by Zinke’s approach. PCR-ALDA assay and Sanger sequencing could coherently identify 12 CALR mutant samples but the Zinke’s Real-time PCR method detected only 10 positive samples out of Jak2 V617F negative cases. With the single use of *Jak2* mutation screening assay (tetra primer assay), only 60% (63/105) of MPN patients were identified, but with the combinatory uses of *Jak2* mutation screening and PCR-ALDA assays, 71.4% (75/105) of patients were diagnosed during hospitalization.

We consider that PCR- ALDA assay might not be able to resolve mutant versus the wild-type alleles of *CALR* if the mutant amplicons differ at less 5 bps from that of wild-type. However, *CALR* mutations that possess deletion or insertion stretches shorter than 5 bps are very infrequent [14]. Thus, we believe that it is seldom for our PCR-ALDA to leak the diagnostics of *CALR* mutations if the clinical samples contain more than 1% of mutant alleles. We also be aware that it is needed to have methodologies that can precisely quantify the burden of *CALR* mutant alleles allowing the physicians to indirectly monitor treatment responses to specific therapies like situations in which the quantitative levels of *Jak2* V617F allele burden were used as the predictive marker for treatment response in PV patients [15, 24, 25]. Nonetheless, in this current study, the quantification of *CALR* mutant alleles has not yet validated therefore, we recommend to utilize the assay for identification of *CALR* mutations but not for quantification of *CALR* mutant alleles.

## Conclusions

In this study, we demonstrated a rapid, low cost and sensitive detection of Calreticulin mutations by a PCR based amplicon length differentiation assay for diagnosis of myeloproliferative neoplasms.

## Additional files

Additional file 1: **Table S1**. Primer pairs utilized in this study. (DOCX 14 kb)

Additional file 2: **Figure S1**. Electropherograms illustrating /CALR/ -type1 and -type 2 mutations. (PNG 1375 kb)

Additional file 3: **Figure S2**. Limit of detection of /CALR/ -type1 and -type 2 mutants by Zinke’s Real-time PCR. (PNG 2205 kb)

## Abbreviations

ALDA: Amplicon Length Differentiation Assay
ARMS: Amplification-refractory mutation system
CALR: Calreticulin
CML: Chronic myeloid leukemia
ET: Essential thombocythemia
Jak2: Janus Kinase 2
*MPL*: Myeloproliferative leukemic
MPN: Myeloproliferative neoplasms
PM: Primary myelofibrosis
PV: Polycythemia vera
