# Supplementary material for: Rapid, low cost and sensitive detection of Calreticulin mutations by a PCR based amplicon length differentiation assay for diagnosis of myeloproliferative neoplasms
Source: BMC Med Genet. 2019 Jun 27;20:115. doi: 10.1186/s12881-019-0819-6 (PMC6598322; doi:10.1186/s12881-019-0819-6)
Supplement: Supplementary file 1 — Table S1. Primer pairs utilized in this study. (DOCX 14 kb) [file 12881_2019_819_MOESM1_ESM.docx]

**Additional file 1: Table 1**

| Primers utilized | Primer Sequences | Purpose |
| --- | --- | --- |
| Tr-Jak2-F | 5’-TCCTCAGAACGTTGATGGCAGTTG-3’ | Outer primer for identification of Jak2-V617F mutation |
| TR-Jak2-R | 5’-TCAGTTTCAAAAATACTTAACTCCTGT-3’ |  |
| Tr-V617F-WT-F | 5’-GCATTTGGTTTTAAATTATGGAGTATTTG-3’ | Inner Wild-type specific primer |
| Tr-V617F-MT-R | 5’-GTTTTACTTACTCTCGTCTCCACATAA-3’ | Inner Mutant specific primer |
| TR-CALR-F | 5’-ACAAATGAAGGACAAACAGGACGA-3’ | To differentiate CALR type -1 (52bp deletion) and a type-2 (5bp insertion) |
| Q-Re-Del-CALR | 5’- CGGGGACATCTTCCTCCTCAT-3’ | To differentiate CALR type -1 (52bp deletion) and a type-2 (5bp insertion) |
