# Supplementary figures and images for: Rapid, low cost and sensitive detection of Calreticulin mutations by a PCR based amplicon length differentiation assay for diagnosis of myeloproliferative neoplasms
Source: BMC Med Genet. 2019 Jun 27;20:115. doi: 10.1186/s12881-019-0819-6 (PMC6598322; doi:10.1186/s12881-019-0819-6)

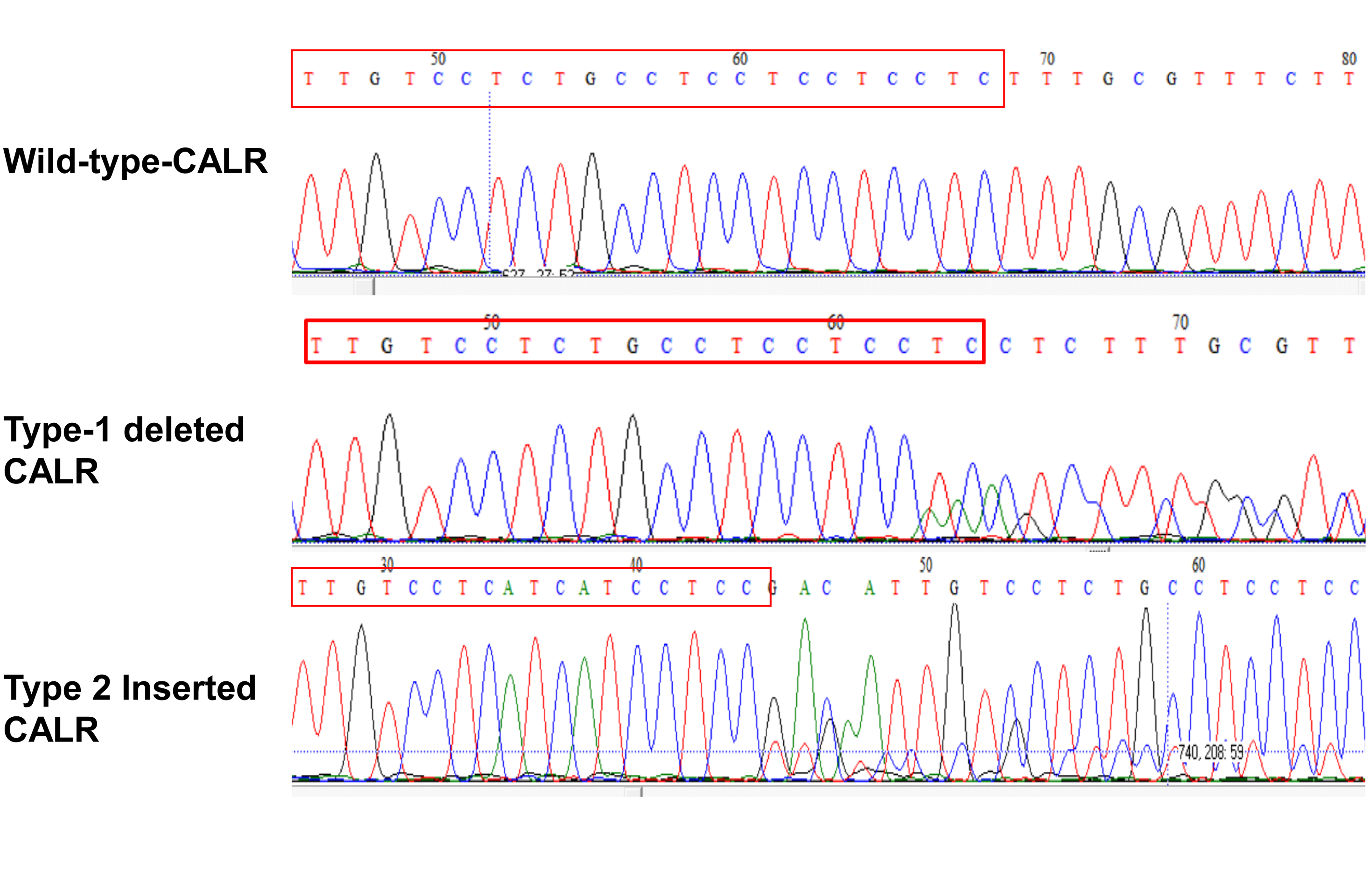

Supplement: Supplementary file 2 — Figure S1. Electropherograms illustrating /CALR/ -type1 and -type 2 mutations. (PNG 1375 kb) [file 12881_2019_819_MOESM2_ESM.png]

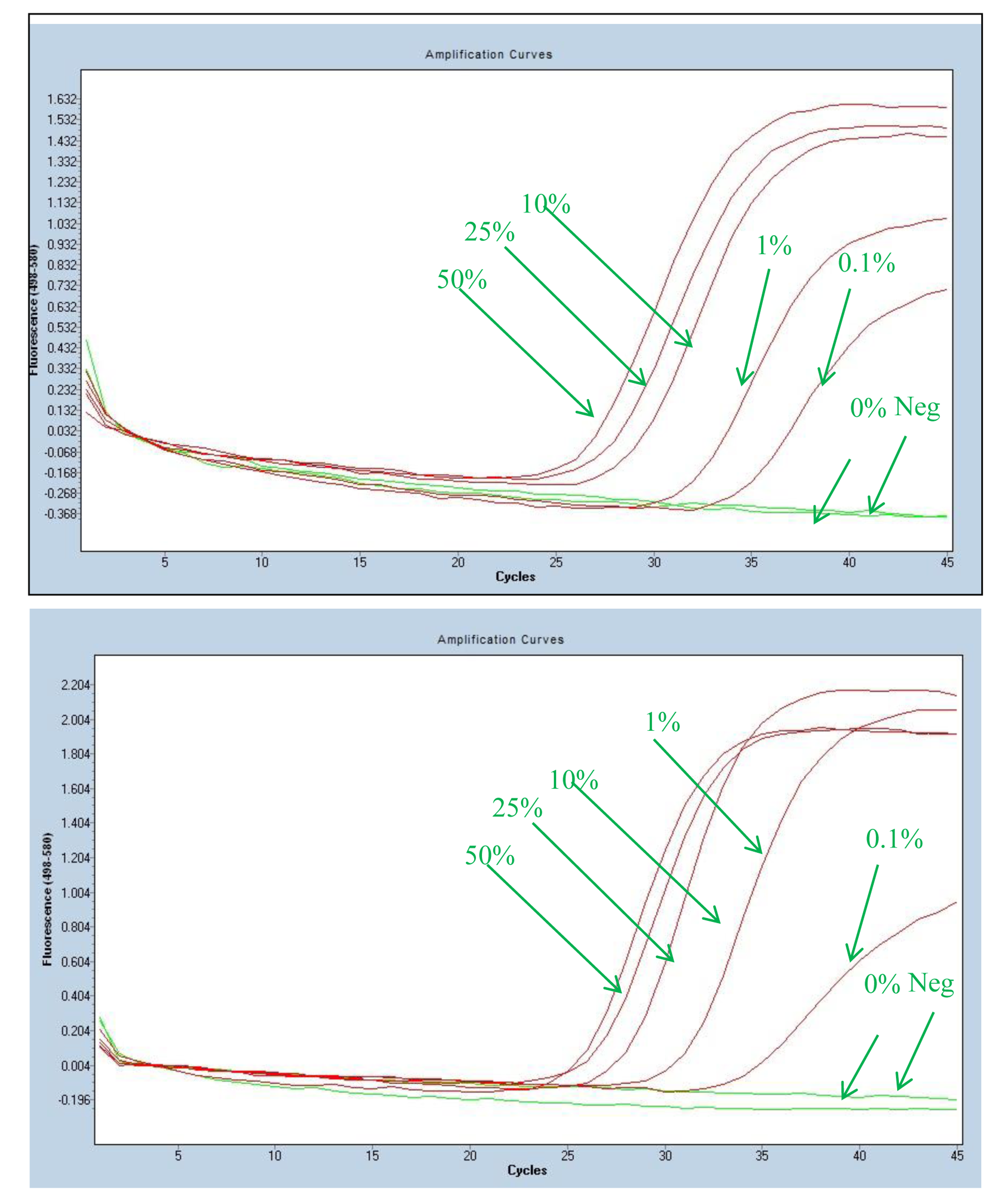

Supplement: Supplementary file 3 — Figure S2. Limit of detection of /CALR/ -type1 and -type 2 mutants by Zinke’s Real-time PCR. (PNG 2205 kb) [file 12881_2019_819_MOESM3_ESM.png]
